# Decreased Weight Loss Following Bariatric Surgery in Patients with Type 2 Diabetes

**DOI:** 10.1007/s11695-022-06350-z

**Published:** 2022-11-02

**Authors:** Eleni Rebelos, Diego Moriconi, Miikka-Juhani Honka, Marco Anselmino, Monica Nannipieri

**Affiliations:** 1 CNR Institute of Clinical Physiology, Postal Code: 56124 Pisa, Italy; 2 Turku PET Centre, University of Turku, Postal Code: 20520 Turku, Finland; 3 Department of Clinical and Experimental Medicine, University of Pisa, Postal Code: 56126 Pisa, Italy; 4 Unit of Bariatric Surgery, AOUP, Postal Code: 56124 Pisa, Italy

**Keywords:** Bariatric surgery, Weight loss, Type 2 diabetes

## Abstract

**Background:**

Bariatric surgery represents the most effective treatment for achieving significant and sustained weight loss. We aimed to assess whether presence of type 2 diabetes (T2D) at baseline, and T2D remission following bariatric surgery affect the weight loss outcome.

**Methods:**

Data of 312 consecutive morbidly obese subjects who underwent bariatric surgery were analysed. Patients underwent either RYGB (77%), or sleeve gastrectomy (23%), and their body weight was followed-up for 1, 2, 3, 4, and 5 years at regular ambulatory visits (*N* = 269, 312, 210, 151, 105, at each year, respectively). T2D remission was assessed according to the ADA criteria.

**Results:**

In the whole dataset, 92 patients were affected by T2D. Patients with T2D were older than patients without T2D (52 ± 9 vs 45 ± 11 years, *p* < 0.0001), but there were no differences in baseline BMI, sex, and type of intervention received. We found that presence of T2D at baseline was associated with smaller weight loss at 1, 2, 3, 4, and 5 years following bariatric surgery (*δ* BMI at 2 years: − 13.7 [7.7] vs − 16.4 [7.3] kg/m^2^; at 5 years − 12.9 [8.8] vs − 16.3 [8.7] kg/m^2^ in patients with T2D vs patients without T2D respectively, all *p* < 0.05). When dividing the patients with T2D in remitters and non-remitters, non-remitters had significantly smaller weight loss compared to remitters (*δ* BMI at 2 years: − 11.8 [6.3] vs − 15.4 [7.8] kg/m^2^; at 5 years: − 8.0 [7.1] vs − 15.0 [7.2] kg/m^2^, non-remitters vs remitters respectively, all *p* < 0.05).

**Conclusions:**

T2D is independently associated to smaller weight loss following bariatric surgery, especially in subjects not achieving diabetes remission.

**Graphical Abstract:**

• Patients with T2D achieve smaller weight loss following bariatric surgery

• When dividing the T2D patients in remitters and non-remitters, non-remitters achieve significantly smaller weight loss compared to remitters

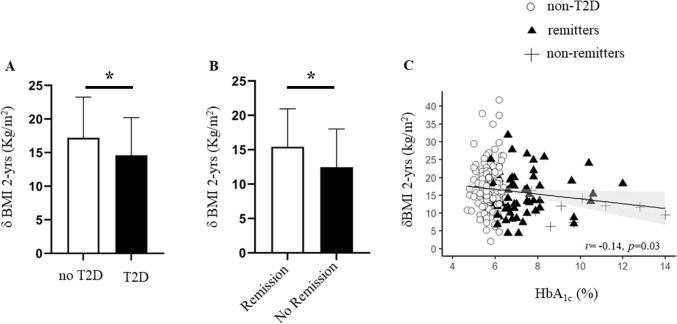

## Introduction

Bariatric surgery is the most efficient treatment to achieve significant and sustained weight loss, and in patients affected by type 2 diabetes (T2D), it consistently induces long-lasting remission of the disease [1]. Following bariatric surgery several alterations occur such as enhanced GLP-1 secretion [2], marked suppression of plasma branched-chain aminoacids [3], increased circulating bile acids [4], and alterations in gut microbiota [5], which have been proposed to contribute to favorable metabolic outcomes. Recently though, in an elegant study by Yoshino and colleagues, the effects of matched weight loss induced by Roux-en-Y gastric bypass (RYGB) or diet alone on a variety of metabolic outcomes such as hepatic, muscle, and adipose tissue insulin sensitivity; β-cell function; the metabolic response to a mixed meal; and 24-h plasma glucose, insulin, and free fatty acid profiles were assessed. They found that both diet and RYGB yielded similar metabolic benefits on the major physiologic factors that regulate glycemic control, suggesting that the beneficial metabolic effects of bariatric surgery can be ascribed solely to weight loss itself, rather than to any weight-loss-independent effects [6]. Since long-term weight loss with lifestyle intervention is typically unsuccessful, the clinical utility of bariatric surgery is unquestionable.

It is now well established that the mechanisms leading to weight loss following bariatric surgery are not to be entirely attributed to decreased nutrient intake, and mild malabsorption of nutrients. Several other mechanisms have been implicated among which the increased secretion of gut-derived hormones with anorectic action, change of alimentary patterns, bile acid, and microbiota adaptations [7]. However, even following bariatric surgery, some patients lose only a small amount of their excess body weight, representing thus treatment failures [8]. The recognition of the predictors of post-bariatric surgery outcomes may thus help in the identification of ideal candidates for this intervention, but may also help setting realistic goals in terms of weight loss outcomes.

There are now several studies assessing the pre-operative determinates of weight loss following bariatric surgery. Among these, age and preoperative BMI are well-known predictors of subsequent weight loss [9]. Post-prandial hypoglycemia is a common complication following bariatric surgery [10], and in a recent study, we have shown that following bariatric surgery, occurrence of post-prandial hypoglycemia associates to a decreased weight loss following bariatric surgery [11]. Presence of co-morbidities before intervention, comprising presence of T2D, has been reported as risk factors for worse outcome following bariatric surgery [9], even though other studies failed to replicate an independent effect of T2D on weight loss following bariatric surgery [12].

In the present study, we assessed whether presence of T2D at baseline predicts weight loss after bariatric surgery. Also, we evaluated whether remission or non-remission from T2D impacts on the post-surgery weight outcome.

## Methods

### Study Design/Study Population

The study population consisted of 312 consecutive subjects who underwent bariatric surgery in our unit and were regularly followed at our outpatient clinic for metabolic control for at least 2 years. Subjects were screened before surgery to evaluate their eligibility for bariatric surgery and to exclude secondary causes of obesity. Further exclusion criteria were the following: (a) previous bariatric surgery, and (b) ongoing severe medical conditions (cirrhosis, end-stage renal failure, malignancy, connective tissue diseases, endocrine diseases). Based on the American Diabetes Association criteria (ADA) (i.e. fasting plasma glucose ≥ 126 mg/dl, 2-h plasma glucose ≥ 200 mg/dl during OGTT, or HbA_1c_ ≥ 6.5%) [13], 25 patients had newly-onset T2D and started treatment with metformin, whereas 67 patients had previously diagnosed T2D at baseline and were treated with metformin only (42%), combination of metformin and dipeptidyl peptidase 4-inhibitors (45%) or basal bolus insulin therapy (13%). All subjects underwent laparoscopic RYGB or laparoscopic sleeve gastrectomy (LSG) at the Bariatric Surgery Unit of our hospital between August 2008 and February 2014. Selection of the intervention was personalized according to the patient’s characteristics (age, BMI, treatment) and co-morbidities (gastroesophageal reflux disease, *H. pylori*, iron/vitamin deficiencies, anemia, non-alcoholic steatohepatitis) after multidisciplinary evaluation (internist and surgeon). The study protocol was approved by the local ethics committee. Prior to enrolment, each participant gave written consent. The study was performed in accordance with the Declaration of Helsinki.

### Follow-Up Visits

**Fig. 1:**
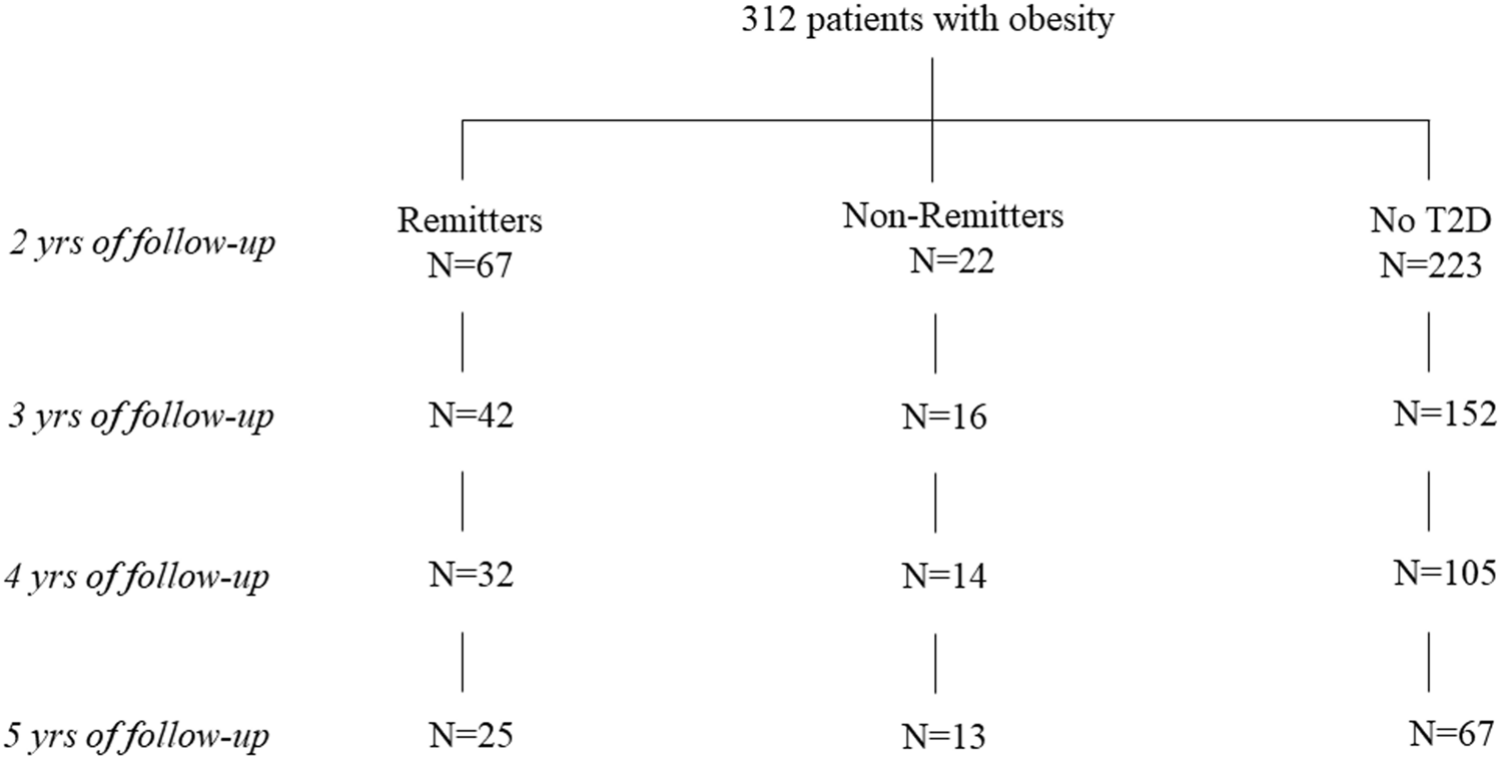
Flowchart showing the number of patients studied at each year of follow-up

After surgery, patients were followed by planned visits at 45 days, three months, six months, and every 6–12 months thereafter, depending on their general status (symptoms, weight loss, metabolic control). During these visits, anthropometric (weight, blood pressure, cardiac frequency) and biochemical measurements were recorded. At 3 years, we had data of 209 patients; at 4 and 5 years of follow-up, data of 151 and 100 patients, respectively, were available (Fig. 1). Postoperatively, percentage of excess body weight loss (EWL) was calculated as follows: [(initial BMI − final BMI)/(initial BMI − 25)] × 100. Failure after bariatric surgery was defined as achieving or maintaining less than 50% of EWL over 24 months [14].

### Remission of T2D

T2D remission was defined as the combination of partial and complete remission according to ADA criteria [15]. In particular, partial remission was defined as HbA_1c_ < 6.5% and fasting glucose 100 − 125 mg/dL, while complete remission was defined as return to fasting glucose < 100 mg/dL and HbA_1c_ < 6.0% for at least 1 year in the absence of pharmacologic therapy or ongoing procedures.

### Statistical Analysis

Continuous variables were expressed as mean ± SD or median [interquartile range (IQR)]. The comparison between continuous variables in patients of different groups was performed by Student’s *t*-test or the Wilcoxon test, as appropriate. The effect of continuous (age, baseline BMI), or nominal (intervention, absence of T2D, T2D remission) variables on weight loss outcomes was assessed with correlation coefficients and 95% confidence intervals, respectively. Three weight loss outcomes were assessed: (*δ* BMI), percentage of weight loss and percentage of EWL. A *p* ≤ 0.05 was considered statistically significant. Statistical analyses were done using JMP version 16.0 (SAS Institute, Cary, NC, USA). The figures were created using *R Studio*.

## Results

### Baseline Data

Of the 312 subjects evaluated, 240 underwent RYGB and 72 LSG. Patients affected by T2D (*N* = 92) were older than those without T2D (52 ± 9 vs 45 ± 11 years, *p* < 0.0001), and also had higher cholesterol and triglycerides values, higher liver enzymes, and worse renal function. On the contrary, baseline BMI was well-matched between the two groups, and there was no difference in the type of bariatric surgery received. The baseline anthropometric and biochemical characteristics of the study participants by presence or not of T2D at baseline are listed in Table 1.

**Table 1:** Baseline anthropometric and biochemical characteristics of the study participants.*

	Patients with T2D		Patients without T2D	*p* value
	Remitters	Non-remitters		
M/W	18/52	6/16	46/174	ns
Age (years)	52 ± 9^§^	55 ± 9	45 ± 11	< 0.0001
BMI (kg/m^2^)	46.1 [8.9]	45.5 [11.2]	46.0 [10.1]	ns
HbA_1c_ (%)	6.9 [1.4]^§^	8.3 [3.6]^#^	5.8 [0.5]	< 0.0001
T2D duration (years)	3 [3]	10 [11]^#^	-	-
Fasting glucose (mg/dL)	121 [53]	149 [157]	87 [15]	< 0.0001
Total cholesterol (mg/dL)	193 [61]	172 [62]	193 [45]	ns
HDL cholesterol (mg/dl)	42 [15]^§^	43 [11]	46 [16]	0.0002
LDL cholesterol (mg/dL)	116 [50]	95 [68]	121 [38]	ns
Triglycerides (mg/dL)	159 [122]^§^	176 [123]	117 [59]	< 0.0001
AST (U/L)	24 [12]^§^	21 [16]	19 [8]	< 0.0001
ALT (U/L)	34 [25]^§^	22 [12]^#^	21 [14]	0.0003
γGT (U/L)	31 [23]^§^	31 [38]	21 [16]	0.0006
MDRD (ml/1.73m^2^/min)	90 [31]^§^	84 [50]	96 [18]	0.01
RYGB/LSG	56/14	9/10^#^	175/48	ns

^*^Entries are mean ± SD, or median [interquartile range], as appropriate. *p* value: comparison between patients without T2D and patients with T2D; ^#^indicates *p* < 0.05 in the comparison between remitters and non-remitters. ^§^Indicates *p* < 0.05 in the comparison between remitters and patients without T2D

### Follow-Up

**Fig. 2:**
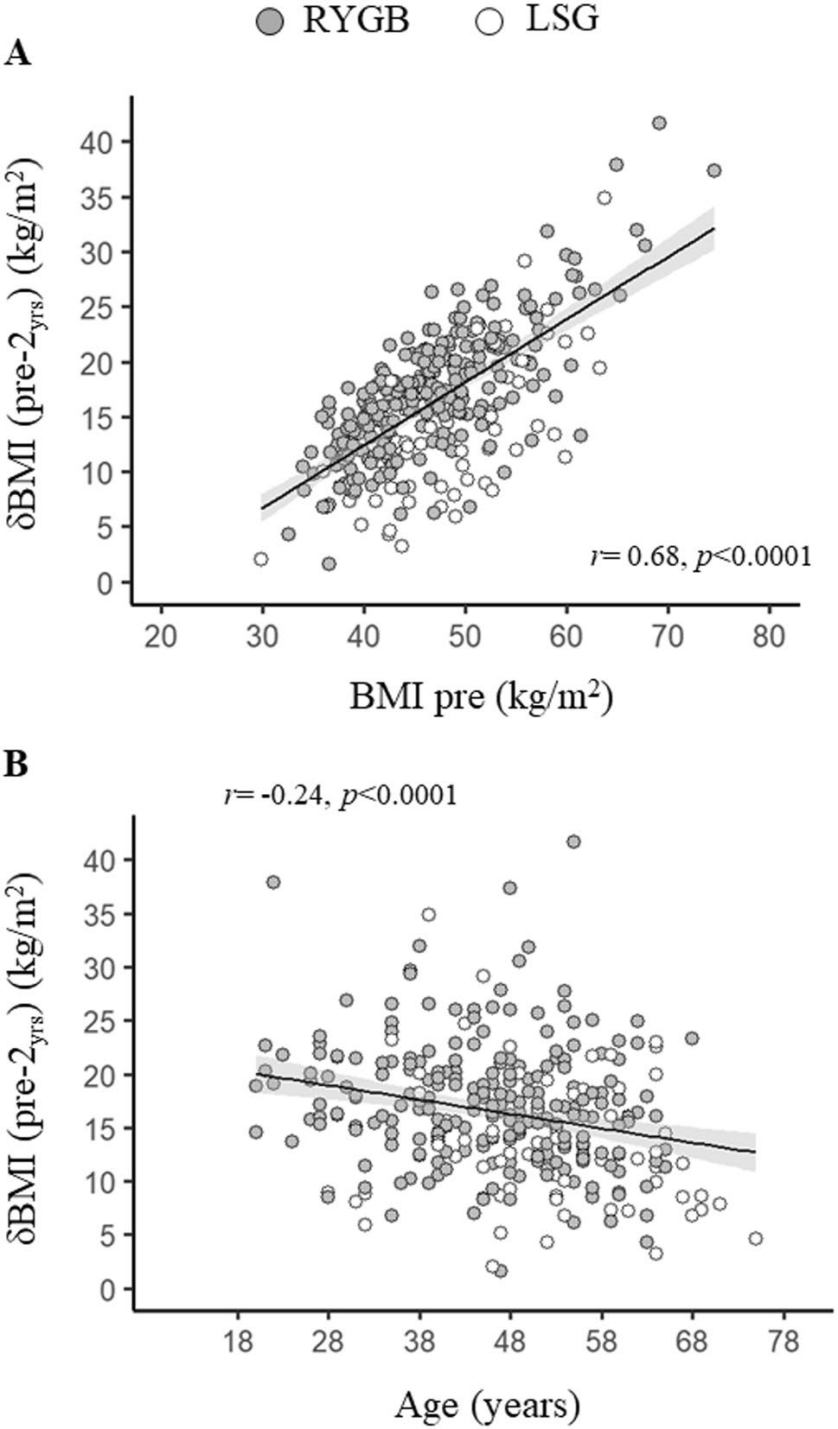
In the pooled data age, baseline BMI (**A**) and age (**B**) were associated with *δ* BMI 2 years of follow-up. Data are shown as RYGB (black circles), or LSG (gray circles)

Following bariatric surgery, patients achieved significant weight loss, losing on average 16 kg/m^2^ at 2 years of follow-up. Age (*r* =  − 0.24, *p* < 0.0001) and baseline BMI (*r* = 0.68, *p* < 0.0001) were associated with *δ* BMI at each time point of the follow-up period (Fig. 2, Table 2). Also, patients who underwent RYGB lost greater amount of body weight compared to patients who received LSG (Table 2). These results held true also in multivariate analyses accounting for each of the other two parameters mentioned respectively (Table 2).

**Table 2:** Correlation coefficients for the effects of age and baseline BMI, or 95% confidence intervals for the effect of RYGB, absence of T2D and remission from T2D on *δ* BMI at 1, 2, 3, 4, and 5 years of follow-up

	*δ* BMI 1 year (*N* = 269)	*δ* BMI 2 years (*N* = 308)	*δ* BMI 3 years (*N* = 210)	*δ* BMI 4 years (*N* = 151)	*δ* BMI 5 years (*N* = 105)
Correlation coefficient					
Age	− 0.24***	− 0.24***	− 0.22**	− 0.19*	− 0.33***
Baseline BMI	0.61***	0.68***	0.68***	0.73***	0.75***
95% confidence interval					
RYGB	0.99–2.49***	1.14–2.71***	0.90–2.90***	0.96–3.72**	1.51–5.34***
Absence of T2D	0.65–2.07***	0.56–2.04***	0.43–2.31**	0.28–2.70*	0.34–3.24*
Non-T2D remission	− 2.72 to 0.29	− 3.51 to − 0.31*	− 4.74 to − 0.72**	− 4.9 to − 0.77**	− 5.97 to − 1.41**

^*^p < 0.05; **p < 0.01; ***p < 0.001

**Fig. 3:**
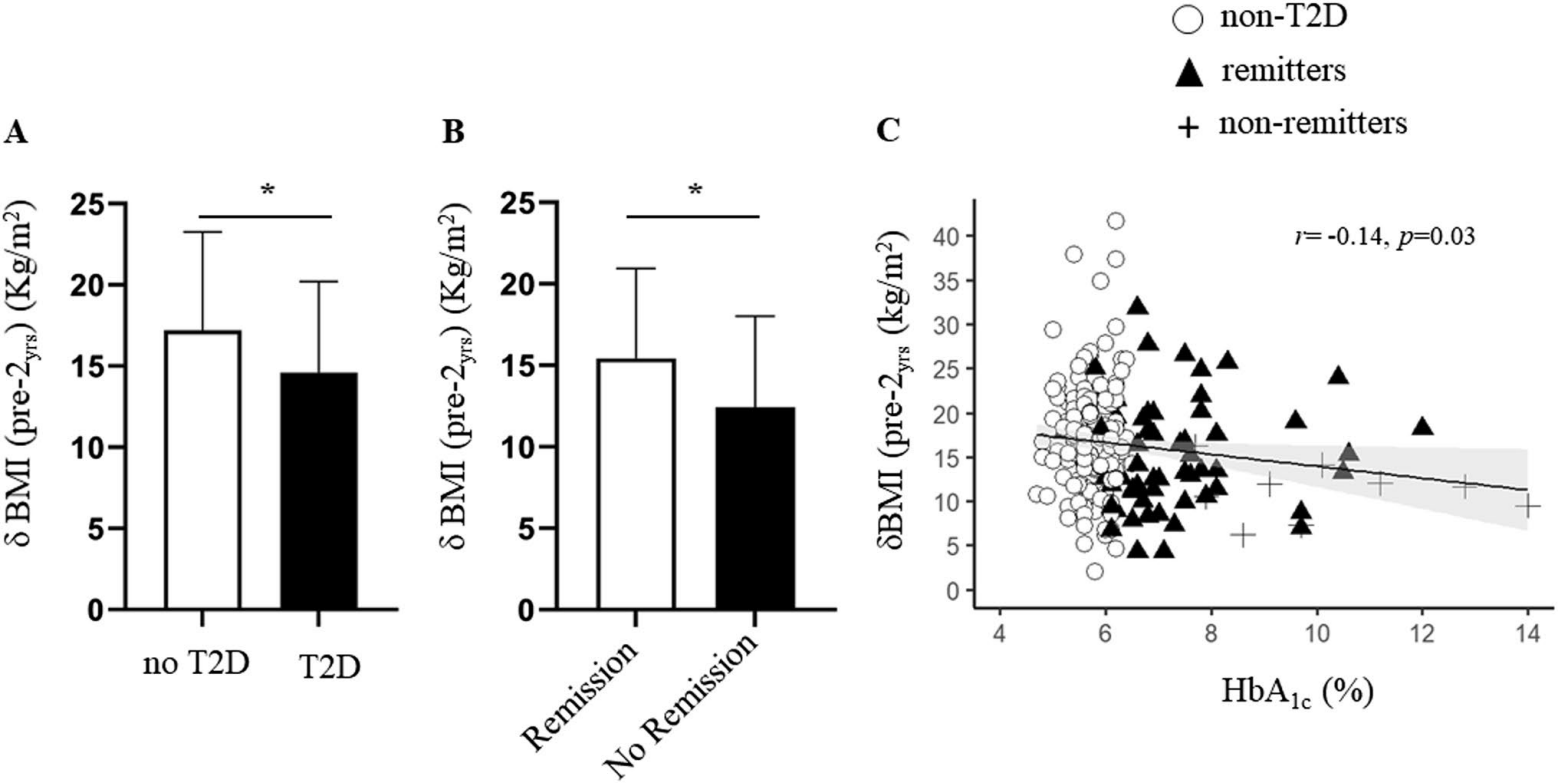
Barplot showing mean *δ* BMI at 2 years of follow-up in patients with T2D and without T2D (**A**). Barplot showing mean *δ* BMI at 2 years of follow-up in patients with remission of T2D and non-remitters (**B**). Baseline HbA_1c_ was inversely related to *δ* BMI at 2 years of follow-up but this association was largely driven by non-remitters (**C**); * *p* < 0.05

In the whole dataset, presence of T2D was associated with a smaller weight loss at 1 and 2 years of follow-up (1 year: − 13.1 [7.7] vs − 15.4 [6.2] kg/m^2^, *p* = 0.0002; 2 year: − 13.7 [7.7] vs − 16.4 [7.3] kg/m^2^, *p* = 0.0006, in patients with and without T2D respectively) (Fig. 3A & Table 3). This result held true also after accounting for age, baseline BMI, and type of surgery (*r*^*2*^ = 0.57, *p* = 0.006, for smaller weight loss in patients with T2D). Furthermore, at longer follow-up of 3, 4, and 5 years, presence of T2D at baseline was associated with smaller weight loss (− 13.1 [8.6] vs − 16.2 [8.1] kg/m^2^, *p* = 0.004; − 14.7 [9.1] vs − 16.7 [8.4] kg/m^2^, *p* = 0.03; vs − 12.9 [8.8] vs − 16.3 [8.7] kg/m^2^, *p* = 0.02; patients with and without T2D respectively at 3, 4, and 5 years follow-up).

**Table 3:** Weight loss outcomes in the three groups.*

	Patients with T2D		Patients without T2D	*p* value
	Remitters	Non-remitters		
*δ* BMI (kg/m^2^)				
1^st^ year	13.2 [8.1]^§^	11.5 [5.1]	15.4 [6.2]	0.0002
2^nd^ year	15.4 [7.8]^§^	11.8 [6.3]^#^	16.4 [7.3]	0.0006
3^rd^ year	15.5 [7.7]	9.7 [5.0]^#^	16.2 [8.1]	0.004
4^th^ year	15.7 [7.9]	9.0 [7.3]^#^	16.7 [8.4]	0.03
5^th^ year	15.0 [7.2]	8.0 [7.1]^#^	16.3 [8.7]	0.02
Percentage of WL (%)				
1^st^ year	29.8 [11.4]^§^	24.0 [9.0]^#^	34.1 [10.5]	< 0.0001
2^nd^ year	32.9 [12.4]^§^	24.5 [16.1]^#^	36.9 [11.6]	< 0.0001
3^rd^ year	32.9 [15.4]	23.3 [12.0]^#^	36.4 [11.9]	0.0006
4^th^ year	32.9 [14.6]	23.4 [0.17]^#^	35.6 [13.2]	0.005
5^th^ year	30.0 [14.6]	19.2 [15.3]^#^	34.5 [12.5]	0.004
Percentage of EWL (%)				
1^st^ year	68.1 [27.9]^§^	53.0 [19.0]^#^	75.3 [26.1]	0.0003
2^nd^ year	72.3 [24.0]^§^	58.3 [32.9]^#^	81.5 [29.2]	0.0002
3^rd^ year	73.7 [24.3]	53.8 [31.7]^#^	79.6 [27.6]	< 0.0001
4^th^ year	70.5 [22.7]	53.1 [47.1]^#^	77.6 [28.2]	0.004
5^th^ year	67.5 [14.7]	51.8 [42.8]^#^	76.3 [31.9]	0.003

^*^Entries are median [interquartile range]. *p* value: comparison between patients without T2D and patients with T2D; ^#^indicates *p* < 0.05 in the comparison between remitters and non-remitters; ^§^indicates *p* < 0.05 in the comparison between remitters and patients without T2D. *WL*, weight loss; *EWL*, excess body weight loss

Since in the present dataset, patients affected by T2D were older and age is an independent predictor of weight loss following bariatric surgery, we performed an ad hoc analysis where in the non-T2D group, we included only patients of matched age (*N* = 122) to the group of patients affected by T2D. When accounting for baseline BMI and type of intervention, presence of T2D was still predicting a smaller weight loss at 2 (*r*^*2*^ = 0.55, *p* = 0.005), 3 (*r*^*2*^ = 0.57, *p* = 0.002), 4 (*r*^*2*^ = 0.58, *p* = 0.009), and 5 years (*r*^*2*^ = 0.63, *p* = 0.04) of follow-up.

In the whole dataset, 35 patients (16 with T2D and 19 without T2D at baseline) achieved at 2-year weight loss smaller than 50% of their EWL, representing thus primary treatment failure. Presence of T2D did not seem to influence occurrence of primary treatment failure, and in a multivariate model accounting for age, T2D, and type of intervention, the only predictor for primary treatment failure was receiving LSG (*p* < 0.0001).

### Remission Vs Non-remission from T2D

**Fig. 4:**
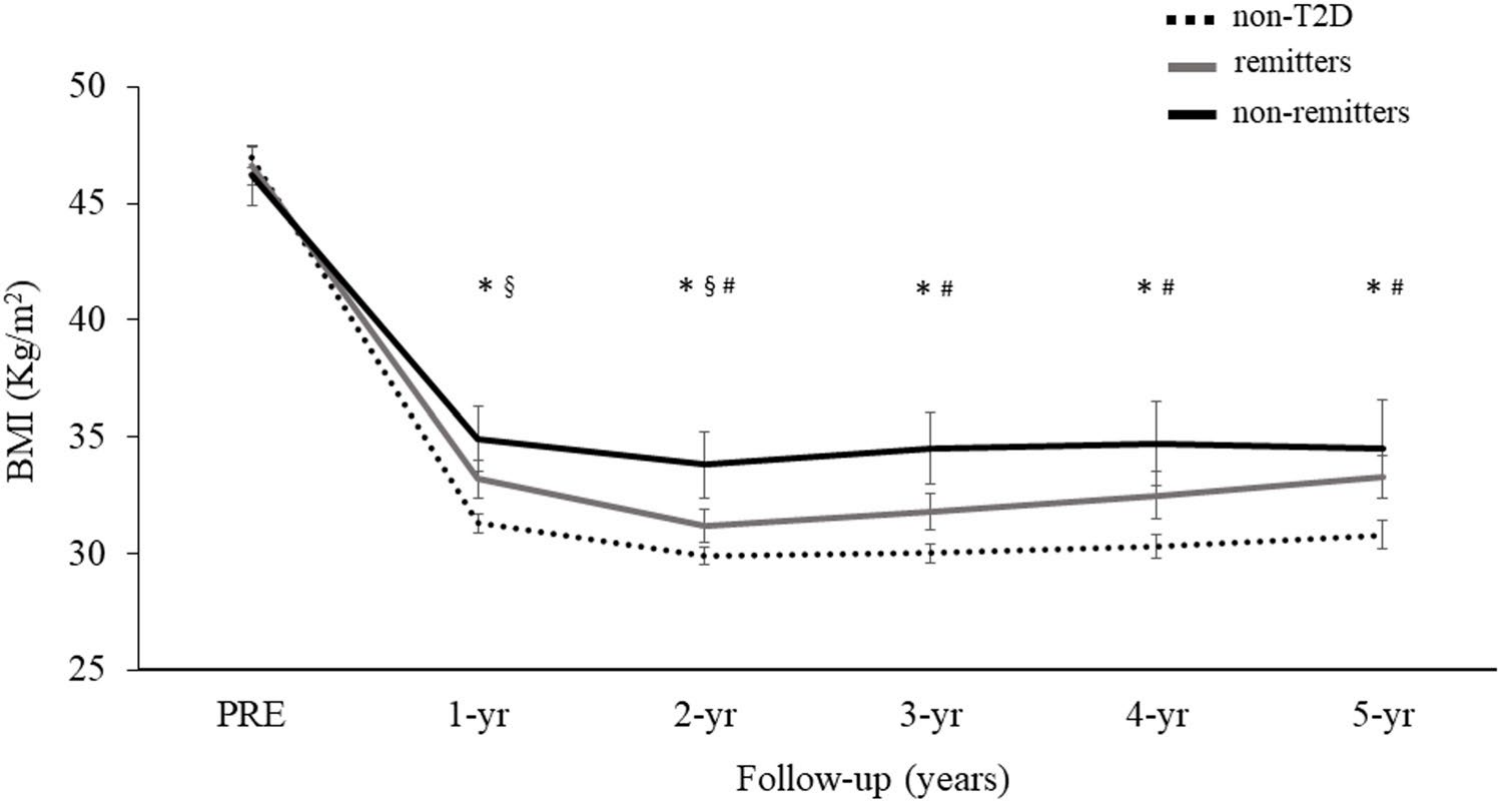
BMI course from baseline over years of follow-up. Black line represents non-remitters, gray line remitters, and dotted line represents patients without T2D. #*p* < 0.05 between non-remitters to remitters; §*p* < 0.05 between remitters and patients without T2D, *p < 0.05 between non-remitters and patients without T2D

We then considered whether patients with T2D at baseline, who following bariatric surgery did not achieve remission (non-remitters, *N* = 22) behaved differently to those who achieved remission within 1 year after surgery (remitters, *N* = 70). We thus divided the dataset in remitters, non-remitters, and patients who did not have T2D. At baseline, non-remitters had higher HbA_1c_ and longer T2D duration compared to remitters, but there was no difference in baseline BMI (Table 1). Table 3 summarizes the weight loss outcomes in the three groups as *δ* BMI, percentage of weight loss, and percentage of EWL. In univariate analysis, at 1 year of follow-up, remitters and non-remitters had achieved similar weight loss (− 13.2 [8.1] vs − 11.5 [5.1] kg/m^2^, *ns*). However, at longer follow-up, non-remitters achieved smaller weight loss compared to remitters: at 2 years (− 11.8 [6.3] vs − 15.4 [7.8] kg/m^2^, *p* = 0.03) (Fig. 3B), at 3 years (− 9.7 [5.0] vs − 15.5 [7.7] kg/m^2^, *p* = 0.008), at 4 years (− 9.0 [7.3] vs − 15.7 [7.9] kg/m^2^, *p* = 0.008), and 5 years (− 8.0 [7.1] vs − 15.0 [7.2] kg/m^2^, *p* = 0.003) (Fig. 4). In line with this, in the pooled data, HbA_1c_ was inversely related to *δ* BMI at 2 years of follow-up, but this association was driven by the non-remitters (Fig. 3C).

In an ad hoc analysis, we then excluded from the dataset the 22 non-remitters, to assess whether there was any difference in weight loss between remitters and patients without T2D. Remitters lost less weight at 2 years of follow-up compared to patients without T2D (− 15.4 [7.8] vs − 16.4 [7.3] kg/m^2^, *p* = 0.04) but there was no significant difference between the two groups in *δ* BMI at 3–5 years of follow-up (Fig. 4).

## Discussion

The main findings of the present study are two: first, morbidly obese patients who are also affected by T2D lose less weight following bariatric surgery, compared to morbidly obese counterparts not affected by T2D. Second, this decreased weight loss in patients with T2D is largely explained by those patients who did not achieve T2D remission after surgery.

Our data are in line with two earlier reports which have shown that presence of T2D predicts a smaller reduction in body weight following bariatric surgery [16, 17]. However, those studies were limited to only 1 year after bariatric surgery; thus, it was not known if this finding was confirmed also in longer follow-up. In a recent large study from the SOS registry, Brissman et al. showed that presence of T2D at baseline was predicting smaller weight loss up to 5 years following bariatric surgery [18]. Our findings are thus in line with the findings reported by Brissman et al. showing that presence of T2D has a negative impact on the short and long-term control of body weight following bariatric surgery.

Moreover, we further elaborate on this finding by demonstrating that when accounting for T2D remission, non-remitters are those who also have smaller weight loss. Remission occurred within 1 year following bariatric surgery and at the first year of follow-up remitters and non-remitters had achieved similar weight loss. The effect of non-remission on weight loss becomes evident from the second year onwards. Of note, that in the present dataset non-remitters had longer duration of T2D and worse glycemic control compared to remitters at baseline, indicating that non-remitters were more ill and with long-standing disease compared to remitters. This is further supported by the correlation between HbA_1c_ and *δ* BMI (Fig. 3C), which showed a non-linear correlation driven by the presence of non-remitters. In a study by Laurino et al. in 140 patients who underwent RYGB, the authors also reported that T2D remission correlated with the nadir and final (5 years of follow-up) body weight [19]. However in that study, the prevalence of T2D in the whole dataset was smaller (18%) compared to ours (29%) resulting in a very small number of patients with T2D at follow-up. Also, it is not clear whether the effect of possible confounders (e.g. age) was accounted for.

As already discussed, from the present analyses emerges clearly that non-remitters achieve smaller weight loss (Fig. 4 & Table 3). On the contrary, our data do not provide a solid conclusion whether also remitters achieve smaller weight loss compared to patients who were not affected by T2D. At 2 years of follow-up, remitters had achieved smaller weight loss compared to patients without T2D, but this effect was not confirmed at longer follow-up. This might be explained by the fact that at longer follow-up our study suffered from relatively large drop-out rates, decreasing thus the sample size and our statistical power to detect smaller effects.

Even though the importance of bariatric surgery in both decreasing body weight [20] and improving a series of comorbidities comprising T2D [20, 21] is unquestionable, our results suggest that the weight loss expectations of patients with T2D following bariatric surgery should be discussed with their clinicians and realistic weight loss goals should be set. This is an important aspect since patients following bariatric surgery may suffer from frustration due to small weight loss, or weight regain [22].

Also, one salient issue to clarify would be whether non-remitters did not achieve T2D remission because they did not lose significant amount of their excess body weight, or whether vice versa the long-lasting course of T2D and the poor glycometabolic control in non-remitters were factors that precluded them from achieving adequate weight loss. The existing data in the present dataset does not allow us to investigate this topic, but the general understanding thus far has been that non-remission from T2D is largely to be attributed to pre-surgery long disease duration and poor glucometabolic control[1], characteristics that were also confirmed in the present dataset (Table 1). Moreover, thus far, and to the best of our knowledge the mechanism(s) underlying the smaller weight loss in morbidly obese patients affected by T2D following bariatric surgery are not known. The understanding of the molecular mechanisms through which T2D is linked to decreased weight loss following bariatric surgery may be of paramount significance, since it could un-mask a link between glucometabolic disorders and weight control which has thus far gone undetected. On the contrary, caloric restriction (e.g. through diets) may not be sufficient to reveal it due to typically smaller weight loss achieved with dietary interventions [23].

It is well established that following bariatric surgery, postprandial GLP-1 secretion is enhanced and this is one of the mechanisms leading to weight loss [24]. Patients with insulin resistance [25] or T2D [26] are characterised by a blunted GLP-1 secretion following a meal. Moreover, we have previously shown that following RYGB patients who did not achieve T2D remission had lower GLP-1 response to a mixed meal compared to remitters [27]. Taken together, a plausible hypothesis might be that one of the mechanisms involved in the smaller weight loss achieved in non-remitters are decreased levels of GLP-1. However, several other mechanisms could be involved. Namely altered secretion of other gastrointestinal hormones and/or of adipokines, a larger suppression of resting energy expenditure, wrong alimentary habits, or altered satiety/reward signals in patients with long-lasting T2D. For instance, recent research lines have shown that the brain may be involved in the control of whole-body homeostasis and weight control [28–31]. In the TULIP study, subjects with central insulin resistance assessed with magnetoencephalography were shown to have worse adherence to lifestyle modification, and smaller weight loss [28], whereas a recent PET study has shown that µ-opioid receptors availability correlates with body weight following bariatric surgery [31]. Thus, whether there are specific brain metabolism alterations in patients with T2D that contribute to decreased weight loss following bariatric surgery merits attention.

Strengths of the present study are the relatively large sample size up to 2 years of follow-up, and the acquisition of data up to 5 years. The inclusion of patients who underwent both RYGB and LSG, the relatively high prevalence of T2D, as also the assessment not only of the baseline T2D status, but also of remission vs non-remission of T2D, and the use of statistical models for controlling for possible confounders are also considerable strengths of the present study. Our study has also limitations. First and foremost, the current data cannot address whether non-remitters do not achieve T2D remission because of smaller weight loss, or whether vice versa persistence of T2D following bariatric surgery leads to decreased weight loss. Second, based on the existing data we cannot assess the mechanisms behind our findings. Moreover, even though the starting sample size consisted of 312 patients with morbid obesity who were followed-up to 2 years, the sample size was decreased with prolonged follow-up, as it often happens in studies following bariatric surgery. As shown in Fig. 1, from the fourth year onwards, patients who did not continue in the follow-up were mainly patients without T2D or patients who had achieved T2D remission. Patients who did not achieve T2D remission had slightly higher follow-up rate, which may be attributable to the fact that these patients required treatment also for T2D. Finally, in the present dataset, relapse of T2D occurred only in few patients at varying time points, not allowing us to separately assess the effect of T2D relapse on body weight.

In conclusion, patients affected by T2D show smaller reduction in body weight following bariatric surgery, and this finding is largely driven by patients who do not achieve T2D remission following bariatric surgery. The understanding of the mechanisms that lead to smaller weight loss in patients with T2D warrants investigation.

## Abbreviations

BMI: Body mass index
EWL: Excess body weight loss
GLP-1: Glucagon-like peptide 1
IFG: Impaired fasting glucose
IGT: Impaired glucose tolerance
IQR: Interquartile range
LSG: Laparoscopic sleeve gastrectomy
MMT: Mixed meal test
OGTT: Oral glucose tolerance test
RYGB: Roux-en-Y gastric bypass
T2D: Type 2 diabetes
WL: Weight loss
